# OrganoidPortal: Web server and single‐cell transcriptome database featuring reference atlases of organoids

**DOI:** 10.1002/ctm2.1794

**Published:** 2024-08-07

**Authors:** Jialin Chen, Hao Yu, Ke Sui, Hai Fang, Xi Zhang, Zheng Wang

**Affiliations:** ^1^ Jinfeng Laboratory Chongqing China; ^2^ Medical Center of Hematology, Xinqiao Hospital of Army Medical University, State Key Laboratory of Trauma and Chemical Poisoning Chongqing Key Laboratory of Hematology and Microenvironment Chongqing China; ^3^ Shanghai Institute of Hematology, State Key Laboratory of Medical Genomics, National Research Center for Translational Medicine at Shanghai Ruijin Hospital, Shanghai Jiao Tong University School of Medicine Shanghai China; ^4^ Bio‐Med Informatics Research Center & Clinical Research Center The Second Affiliated Hospital, Army Medical University Chongqing China

Dear Editor,

The emergence and progression of organoids, stem cell‐derived 3D culture systems, have presented valuable prospects for investigating physiological development and diseases.^1^, ^2^ The analysis of transcriptome and regulatory landscape at single‐cell resolution within organoids has shed light on the exploration of novel cell types, pathophysiological mechanisms, drug targets, and biomarkers.^3^ Here, we present OrganoidPortal (http://organoidportal.jflab.ac.cn:18083/app/organoidportal), a user‐friendly, up‐to‐date and dedicated data resource and versatile web server for the efficient analysis, interpretation, transcriptome profiling and evaluation of organoids. It addresses the deficiencies in existing databases by providing the most comprehensive functionality within its field (Table S1).

OrganoidPortal comprises single‐cell transcriptome profiles of 5 565 768 cells from 28 tissues, 1749 samples and four species, obtained from 280 published studies retrieved from various resources and archives (Figure 1A,B and Figure S1A). In the data preprocessing phase, we conducted several essential steps, including the elimination of doublets and low‐quality cells, the removal of discarded genes, as well as normalization and standardization of the refined dataset (Figure 1B and Figure S1A). OrganoidPortal conducts a standardized pipeline for cell clustering, cell type annotation, developmental trajectory prediction, ligand‐receptor interaction, pathway enrichment, gene regulation, and copy number variation (CNV) analyses (Figure 1B and Figure S1A). Furthermore, functional modules of transcriptome data analyses, reference atlases, comparative molecular profiling, and tools for de novo analysis are provided in this robust web server where users can click on the module name in the navigation bar at the top of the homepage to get to each module (Figure 1C and Figure S2A).

**FIGURE 1:**
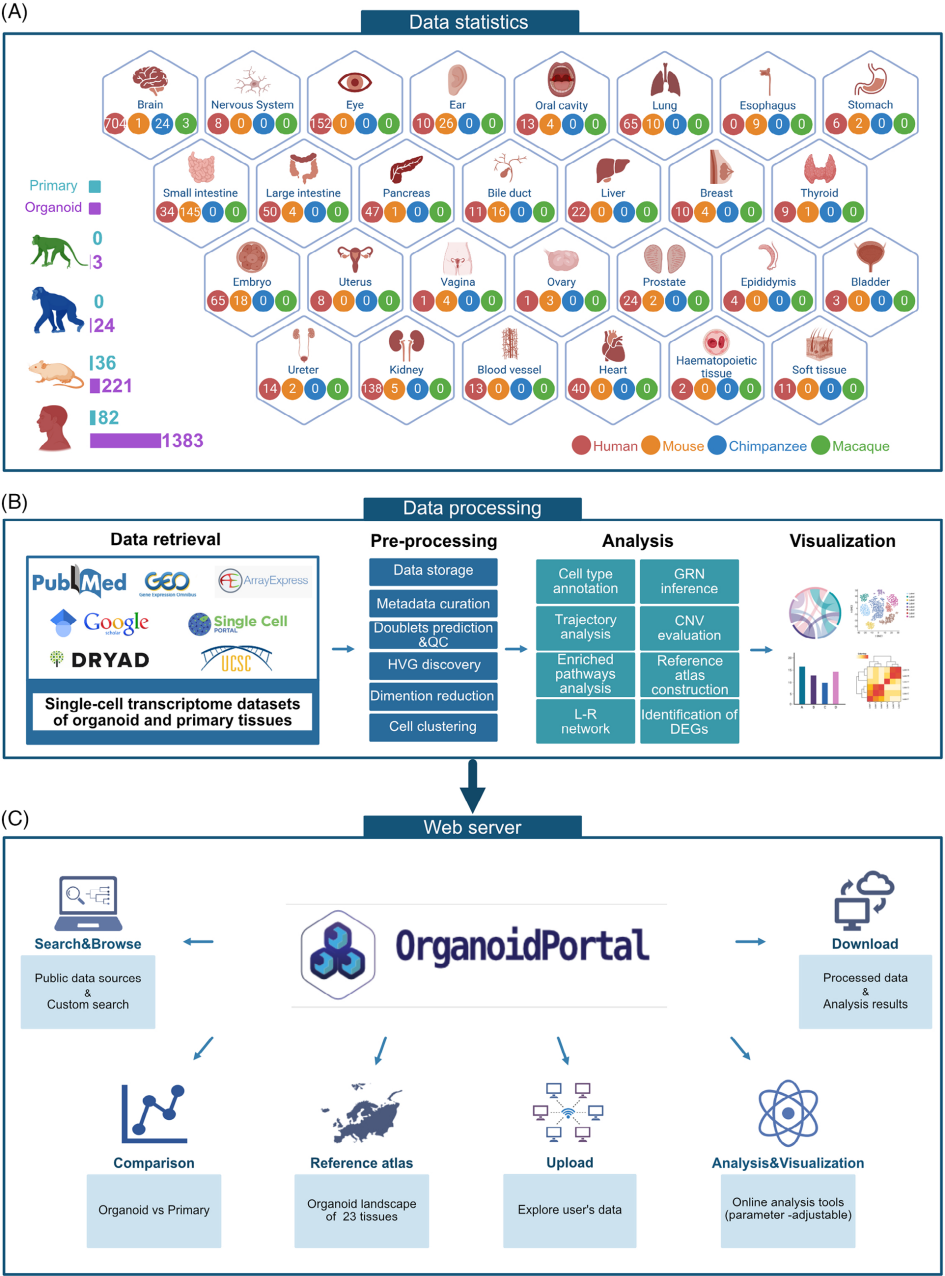
Overview of OrganoidPortal. (A) Statistics of datasets in OrganoidPortal which consolidates 1749 single‐cell transcriptome datasets of organoids and their matching tissues from four species and 28 tissues. (B) The workflow showing the construction of OrganoidPortal, consisting of data retrieval, pre‐processing, analysis and visualization. (C) The framework of OrganoidPortal modules, including data management, interactive visualization, comparison between primary samples and organoids, reference atlases, and a one‐stop analysis module.

OrganoidPortal has devised a data storage and management module called Project Catalog, which meticulously logs manually curated meta‐information, including sample information such as the source, species, tissue and cell count along with DOI link, title, and abstract of original publication (Figure S2B). The Exploration module facilitates the visualization and querying of six aspects of data analysis for each dataset, namely Clusters Visualization, Developmental Trajectory, Pathway Enrichment, Cell‐cell communication, Transcription Factor, and CNV (Figure 2A). The six analysis interfaces present cell clusters and annotation (Figure S3A), trajectory inference (Figure S3B), enriched pathways across cell types (Figure S3C), cell‐cell interaction mediation by ligand‐receptor complexes (Figure S4A), transcription factor activities in different cell clusters (Figure S4B), and assessment of chromosomal CNV using inferCNVpy^4^ (v0.3.0) (Figure S5A).

**FIGURE 2:**
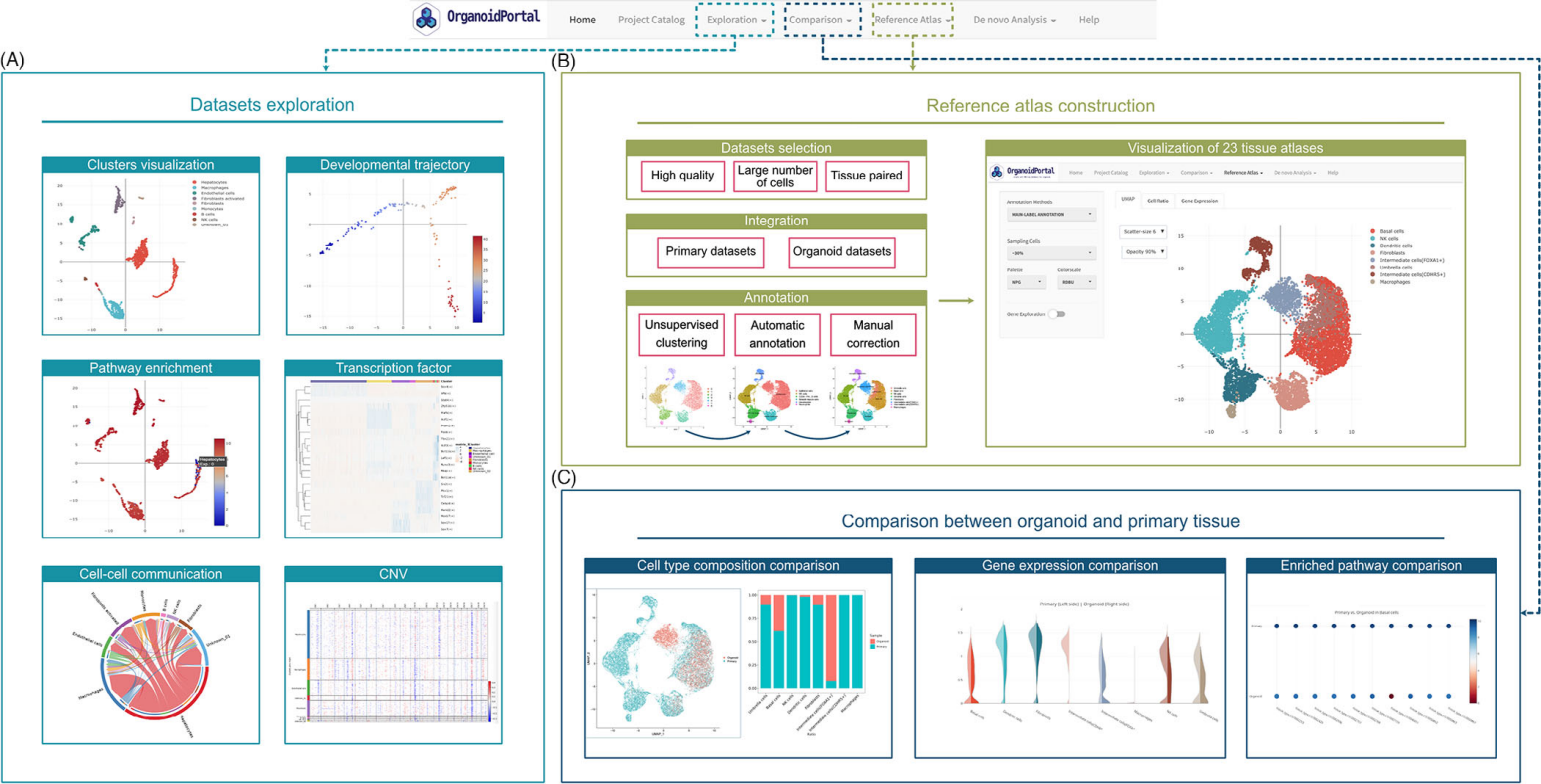
Modules of functional analyses in OrganoidPortal. (A) For each dataset in the database, OrganoidPortal conducts a standardized pipeline for comprehensive downstream analysis and visualization, including cell clustering, cell type annotation, developmental trajectory prediction, ligand‐receptor interaction, pathway enrichment, transcriptional factor regulation, and copy number variation analyses. (B) Reference atlases module of OrganoidPortal. The diagram (left) outlined the process used in the construction of the high‐quality reference atlases, including dataset screening, data integration and cell type annotation. The UMAP plot (right) displays an example of a reference atlas. (C) Visualization of cell composition, gene expression comparison and enriched pathways between organoids and matched primary tissue in the “Comparison” module of OrganoidPortal.

Aiming to capture the broad spectrum of cell states, we built reference atlases by integrating high‐quality amalgamated primary tissue and organoid datasets derived from normal human or mouse samples for 23 tissues (Figure 2B and Figure S1B). Following the removal of low‐quality cells through data preprocessing, the datasets underwent normalization using the sctransform^5^ function from the Seurat^6^ (v4.3.0) software. Subsequently, the top 3000 highly variable genes (HVGs) were chosen for each sample. Once features suitable for integration were identified, the normalized samples were integrated using the “PrepSCTIntegration” function. The correction of batch effects was achieved through the utilization of the IntegrateData function, which incorporated the computed anchor set. Utilizing a fusion of organoids and corresponding primary samples in a healthy state, OrganoidPortal successfully produced cell atlases that were automatically annotated, and subsequently refined through manual correction using original annotations from publications, and standardized annotations based on HVGs (Figure S1B). Users can examine reference atlas annotations as well as expression levels of genes of interest by clicking on the “EXPLOR” button, while reference datasets are available for download by clicking on the “GET” button (Figure S5B).

In order to fully harness the potential of organoids as a promising avenue for advancement in development, disease research, and drug development, it is crucial to generate organoids with sufficient tissue organization. Therefore, OrganoidPortal offers a Comparison module, which enables comprehensive molecular profiling comparisons between organoids at various developmental stages or under diverse cultural conditions, alongside corresponding consolidated primary tissue samples (Figure 2C and Figure S1C). Utilizing UMAP, we visually present the patterns exhibited by organoids and primary tissue cells, while a bar plot illustrates the percentage of organoids and primary tissue cells within each cell type (Figure 2C). In addition, OrganoidPortal provides heatmap and gene expression statistics queries, which assist in visualizing differentially expressed genes (DEGs) in selected cell types, together with mechanistic insight in terms of pathway enrichment analysis (Figure 2C and Figure S5C).

We have developed a module for de novo analysis that enables users to upload their own single‐cell RNA sequencing (scRNA‐seq) datasets of organoids to conduct a thorough analysis, with visualized outputs conveniently downloadable (Figure 3A and Figure S1D). By uploading their scRNA‐seq datasets formatted as H5AD or RDS, users can obtain comprehensive outcomes analyzed using our online standardized workflow. The analyses include the following components: (1) The “Cell Mapping” functional module, as the initial step of data analysis, uses methods of WNN in Seurat or scANVI in scvi‐tools to map users’ datasets onto the build‐in reference atlases with default parameters settings to predict cell types (Figure 3B); (2) The “Gene Exploration” functional module enables the visualization of distinctive gene expression levels across different cell types using UMAP and violin graphs (Figure 3C); (3) Trajectory inference using Monocle2^7^ or Slingshot^8^ of each identified cell type is demonstrated in the “Development Trajectory” module. It also allows users to observe expression levels of genes along pseudotime to better understand differentiation trajectories (Figure 3D); (4) The “Pathway Enrichment” module displays the results of signalling pathway enrichment analysis, calculated using the top 2000 high variable genes with clusterProfiler^9^ (Figure 3E); (5) Using a statistically robust mean method “trimean” in CellChat,^10^ the “Cell‐cell Communication” module infers, analyzes and visualizes the significant ligand‐receptor networks for cell‐cell interaction (Figure 3F).

**FIGURE 3:**
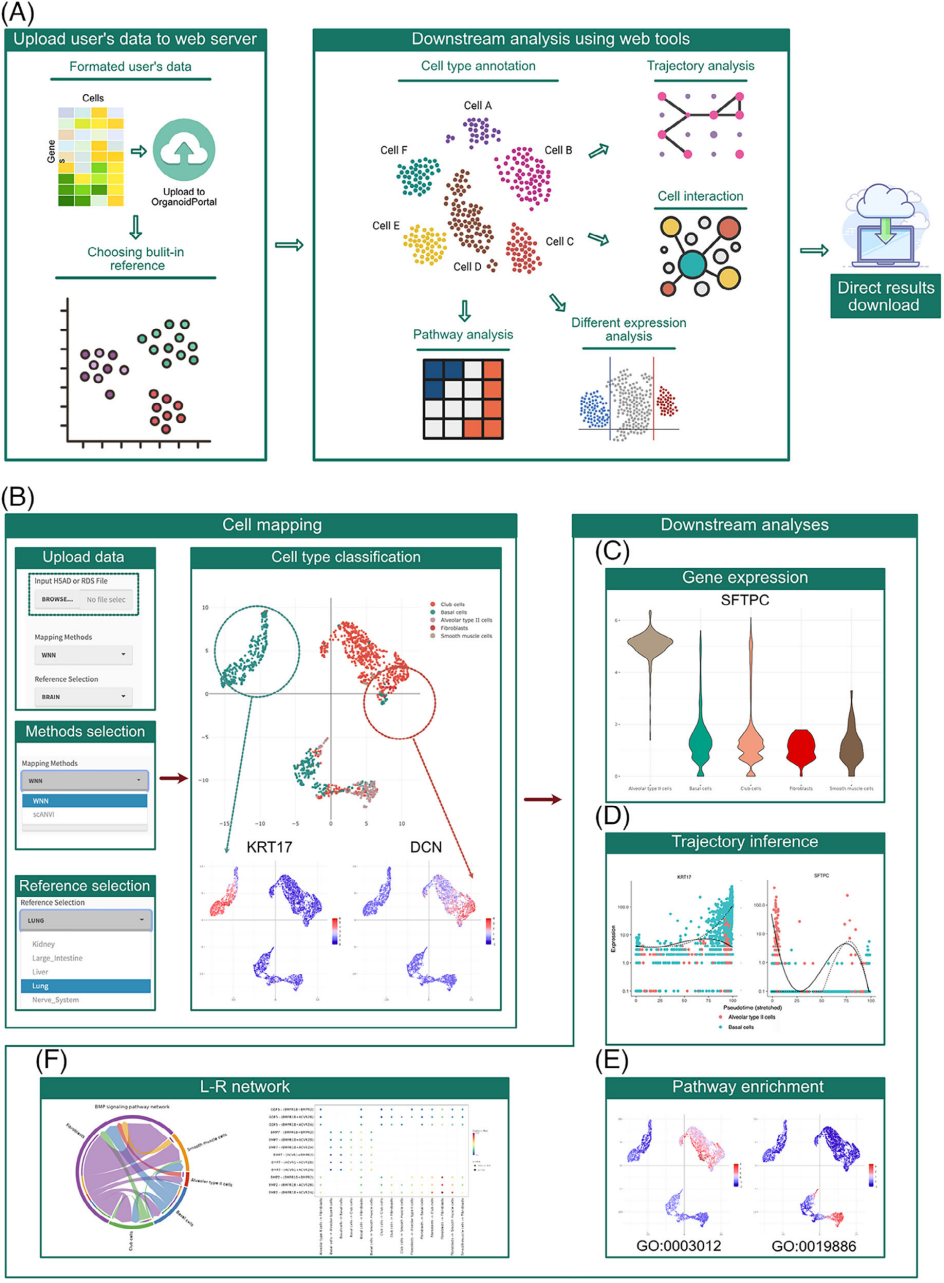
Workflow and presentation of online analysis tools for user data. (A) Overview of online data analysis workflow. Users can upload their data in H5AD or RDS format to OrganoidPortal for subsequent analysis. (B) The web server offers high‐quality built‐in reference atlases of 23 tissues for cell mapping and annotation. The results of cell type classification and downstream analysis can be directly downloaded. An example of all‐in‐one online analyses showing parameters and outcomes for cell mapping and annotation, (C) expression levels of differentially expressed genes, (D) DDRTree plot of developmental inference and marker gene expression along pseudotime trajectory, (E) enriched pathways displayed by UMAP and (F) a chord plot illustrating ligand‐receptor interaction for cell‐cell communication analysis.

Taken together, OrganoidPortal functions as a specialized data repository that facilitates the collection of contemporary scRNA sequencing datasets. It also provides a standardized pipeline for the analysis of transcriptome data pertaining to organoids and their corresponding primary tissues. Additionally, OrganoidPortal enables the interpretation of these datasets. OrganoidPortal is equipped with modules that effectively facilitate the comparative analysis of organoids derived from various developmental stages or cultured under different conditions. As a robust platform, OrganoidPortal empowers users to employ a standardized workflow for the interpretation of their own datasets and to juxtapose them with other research endeavours. This web‐based platform serves as an invaluable resource for researchers, empowering them to unlock the potential of organoids as a promising platform for translational applications.

## AUTHOR CONTRIBUTIONS

Zheng Wang, Xi Zhang and Hai Fang conceptualized and supervised the project. Jialin Chen, Hao Yu and Ke Sui constructed the OrganoidPOrtal web server, built the project website, and created an online tutorial. Ke Sui and Jialin Chen authored the manuscript and curated all figures. All authors reviewed and approved the final manuscript.

## CONFLICT OF INTEREST STATEMENT

The authors declare no conflict of interest.

## FUNDING INFORMATION

This study was supported by Science and Technology Innovation Key R&D Program of Chongqing [CSTB2023TIAD‐STX0001], National Key R&D Program of China [2022YFA1103300], National Natural Science Foundation of China [82020108004], Translational Research Grant of NCRCH [2020ZKZC02], Youth Talent Development Program from Second Affiliated Hospital, Army Medical University [2022YQB014], National Natural Science Foundation of China [32170663] and Innovative Research Team of High‐Level Local Universities in Shanghai.

## ETHICS STATEMENT

This study exclusively utilized publicly accessible datasets.

## PATIENT CONSENT STATEMENT

We acquired written informed consent for publication.

## Supporting information

Supporting Information

## Data Availability

All data, including data sources and the online website, are freely accessible at http://organoidportal.jflab.ac.cn:18083/app/organoidportal, and no login is required.

## References

[1] Huch M , Dorrell C , Boj SF , et al. In vitro expansion of single Lgr5+ liver stem cells induced by Wnt‐driven regeneration. Nature. 2013;494(7436):247‐250. doi:10.1038/nature11826 23354049 PMC3634804

[2] Nikolaev M , Mitrofanova O , Broguiere N , et al. Homeostatic mini‐intestines through scaffold‐guided organoid morphogenesis. Nature. 2020;585(7826):574‐578. doi:10.1038/s41586-020-2724-8 32939089

[3] Krieger TG , Le Blanc S , Jabs J , et al. Single‐cell analysis of patient‐derived PDAC organoids reveals cell state heterogeneity and a conserved developmental hierarchy. Nat Commun. 2021;12(1):5826. doi:10.1038/s41467-021-26059-4 34611171 PMC8492851

[4] Patel AP , Tirosh I , Trombetta JJ , et al. Single‐cell RNA‐seq highlights intratumoral heterogeneity in primary glioblastoma. Science. 2014;344(6190):1396‐1401. doi:10.1126/science.1254257 24925914 PMC4123637

[5] Hafemeister C , Satija R . Normalization and variance stabilization of single‐cell RNA‐seq data using regularized negative binomial regression. Genome Biol. 2019;20(1):296. doi:10.1186/s13059-019-1874-1 31870423 PMC6927181

[6] Hao Y , Hao S , Andersen‐Nissen E , et al. Integrated analysis of multimodal single‐cell data. Cell. 2021;184(13):3573‐3587. e29 doi:10.1016/j.cell.2021.04.048 34062119 PMC8238499

[7] Trapnell C , Cacchiarelli D , Grimsby J , et al. The dynamics and regulators of cell fate decisions are revealed by pseudotemporal ordering of single cells. Nat Biotechnol. 2014;32(4):381‐386. doi:10.1038/nbt.2859 24658644 PMC4122333

[8] Street K , Risso D , Fletcher RB , et al. Slingshot: cell lineage and pseudotime inference for single‐cell transcriptomics. BMC Genomics. 2018;19(1):477. doi:10.1186/s12864-018-4772-0 29914354 PMC6007078

[9] Yu G , Wang LG , Han Y , He QY . clusterProfiler: an R Package for Comparing Biological Themes Among Gene Clusters. OMICS. 2012;16(5):284‐287. doi:10.1089/omi.2011.0118 22455463 PMC3339379

[10] Jin S , Guerrero‐Juarez CF , Zhang L , et al. Inference and analysis of cell‐cell communication using CellChat. Nat Commun. 2021;12(1):1088. doi:10.1038/s41467-021-21246-9 33597522 PMC7889871

