# Supplementary material for: OrganoidPortal: Web server and single‐cell transcriptome database featuring reference atlases of organoids
Source: Clin Transl Med. 2024 Aug 7;14(8):e1794. doi: 10.1002/ctm2.1794 (PMC11306279; doi:10.1002/ctm2.1794)
Supplement: Supplementary file 1 — Supporting Information [file CTM2-14-e1794-s001.docx]

**Supplementary Materials**

**OrganoidPortal: web server and single-cell transcriptome database featuring reference atlases of organoids**

Jialin Chen^2#^, Hao Yu^2#^, Ke Sui^1,2#^, Hai Fang^4*^, Xi Zhang^1,2*^, Zheng Wang^1,2,3*^

^1^ Medical Center of Hematology, Xinqiao Hospital of Army Medical University; State Key Laboratory of Trauma and Chemical Poisoning; Chongqing Key Laboratory of Hematology and Microenvironment, Chongqing 400037, China.

^2^ Jinfeng Laboratory, Chongqing, 401329, China.

^3^ Bio-Med Informatics Research Center & Clinical Research Center, The Second Affiliated Hospital, Army Medical University, Chongqing 400037, China.

^4^ Shanghai Institute of Hematology, State Key Laboratory of Medical Genomics, National Research Center for Translational Medicine at Shanghai, Ruijin Hospital, Shanghai Jiao Tong University School of Medicine, Shanghai200025, China

#These authors contributed equally.

* These authors jointly supervised this work.

Correspondence to:

Zheng Wang, email: biowz@mail.ustc.edu.cn; biowz1010@tmmu.edu.cn

Xi Zhang, email: zhangxxi@sina.com

Hai Fang, email: fh12355@rjh.com.cn

**Materials and methods**

***Data collection and screening***

A thorough investigation was conducted by employing an extensive search strategy incorporating various keywords such as "single cell RNA-seq", "single cell sequencing", "single cell transcriptome", and "scRNA-seq" in conjunction with terms like "organoid", "iPSC derived organ", and "ESC derived organ" along with the specific name of the tissues. This comprehensive approach resulted in the collection of a total of 1,749 single-cell RNA-seq samples encompassing both organoids and their corresponding primary samples. These samples encompassed 1,465 human samples, 257 mouse samples, 24 chimpanzee samples, and 3 macaque samples, originating from primarily 28 distinct tissues (Table S2). All the raw sequencing data were retrieved from reputable databases such as Gene Expression Omnibus (GEO)^1^ and ArrayExpress^2^, and stored in the "Project Catalog" module. The metadata encompasses essential information such as the source, species, tissue, and cell counts. Additionally, the DOI link, title, and abstract of original publication is furnished as article information.

***Data preprocessing***

The quality control and normalization procedures for each scRNA-seq sample were conducted uniformly, adhering to established best practices as documented in published literature.^3^ Specifically, the cells included less than 500 genes (min.features = 500) and genes that were expressed in less than 3 cells (min.cells = 3) were excluded. The sequencing datasets were processed using the R package Seurat (v4.3.0) to generate a standardized data format as SeuratObj. Subsequently, cells containing over 5% of transcripts from mitochondrial genes or cells with more than 5,000 differentially expressed genes were excluded. To normalize the data after quality control, the global-scaling normalization method "LogNormalize" was employed. Following K-Nearest Neighbors (KNN) network inference, Louvain clustering was performed using the top 10 principal components (PCs) with a resolution of 2.0.

***Cell type annotation***

We utilized the R package SingleR (v2.0.0) for annotating scRNA-seq data obtained from human, chimpanzee, monkey, and mouse samples. For human, chimpanzee, and monkey samples, we selected the HumanPrimaryCellAtlasData (HPCA) as reference, while for mouse samples, we referred to MouseRNAseqData.

***Trajectory inference***

Monocle2 (v2.26.0)^4^ and Slingshot (v2.8.0)^5^ were utilized on lineages with more than 50 cells to estimate lineage differentiation. DDRTree plot was constructed for each sample using Monocle2. Subsequently, Monocle2 and Slingshot were utilized to jointly establish the pseudotime trajectory of each lineage. The default values were assigned to all parameters involved in this procedure.

***Pathway enrichment***

OrganoidPortal performed gene functional enrichment analysis at single-cell resolution according to our previous method.^3,6^ The initial step involved the application of gene-set enrichment analysis to identify categories of input genes and generated the corresponding annotated gene ontology (GO) terms. The background value was determined by comparing the number of genes in a single cell to the total number of genes in the population. At the individual cell level, the foreground value was calculated by comparing the number of genes in each pathway to the total number of genes in the population. The incorporation of clusterProfiler(v4.6.2)^7^ facilitated the execution of the primary stages of gene-set enrichment analysis. Specifically, we used the FindVariableFeatures function in Seurat (version 4.3.0) to extract the top 2000 highly variable genes (HVGs). The R package clusterProfiler was then utilized to enrich the GO pathways of the HVGs, with both the “pvalueCutoff” and “qvalueCutoff” set at 0.01. P-values were adjusted using the Benjamini−Hochberg (BH) multiple testing correction method.

***Cell-cell communication***

The construction of the ligand-receptor (L-R) interaction network was accomplished through CellChat (v1.6.1)^8^, which enables the inference of communication patterns among distinct cell types. Specifically, the L-R pairs that are expressed in less than 15 cells within cell clusters were disregarded. A permutation test was conducted with a p-value threshold of less than 0.05 deemed as statistically significant.

***Regulatory network inference***

PySCENIC (v0.12.1)^9^ was used to infer regulons for each cell type in OrganoidPortal datasets. It applied GENIE3, cisTarget, and AUcell algorithms to identify transcription factors, their targets, and activities. The regulon activities were visualized by UMAP and violin plot.

***Copy number variance***

We utilized infercnvpy (v0.3.0)^10^ to identify abnormal cells based on copy number variations (CNV) and aneuploid scores. The average CNVs of all cells were calculated and applied as control. The inferCNV analysis was performed with parameters including denoise, default hidden Markov model (HMM) settings, and a value of 0.1 for “cutoff”. To reduce the false-positive CNV cells, the default Bayesian latent mixture model was implemented to identify the posterior probabilities of the CNV variation in each cell with the value of 0.5 as threshold.

***Comparison between organoid and primary samples***

In the “Comparison module”, users have the option to choose species and tissue pairs for the purpose of examining differentially expressed genes (DEGs) and exploring a comprehensive overview. This includes access to tables presenting statistical information on DEGs, as well as expression levels of enriched pathways. Specifically, to identify DEGs, we utilized the FindAllMarkers function in Seurat (version 4.3.0) with the parameter “test.use = 'wilcox'”. Genes with an average log fold-change (avg_logFC) greater than 0.5 and an adjusted p-value less than 0.05 were selected for further analysis.

***Reference atlas construction***

The construction of high-quality reference atlases was previously described. The reference atlas module provides essential details about reference atlases, including species, tissue, and cell counts. Additionally, users can download accessible raw data by clicking the “GET” button. The “Explore” button facilitates access to visualization of cell component, ratio of cell clusters and specific gene expression levels in the cell atlas.

**Reference**

1. Edgar R. Gene Expression Omnibus: NCBI gene expression and hybridization array data repository. *Nucleic Acids Research*. 2002;30(1):207-210. doi:10.1093/nar/30.1.207

2. Parkinson H. ArrayExpress--a public repository for microarray gene expression data at the EBI. *Nucleic Acids Research*. 2004;33(Database issue):D553-D555. doi:10.1093/nar/gki056

3. Wang Z, Chai C, Wang R, et al. Single‐cell transcriptome atlas of human mesenchymal stem cells exploring cellular heterogeneity. *Clinical & Translational Med*. 2021;11(12):e650. doi:10.1002/ctm2.650

4. Trapnell C, Cacchiarelli D, Grimsby J, et al. The dynamics and regulators of cell fate decisions are revealed by pseudotemporal ordering of single cells. *Nat Biotechnol*. 2014;32(4):381-386. doi:10.1038/nbt.2859

5. Street K, Risso D, Fletcher RB, et al. Slingshot: cell lineage and pseudotime inference for single-cell transcriptomics. *BMC Genomics*. 2018;19(1):477. doi:10.1186/s12864-018-4772-0

6. Yu H, Wang Y, Zhang X, Wang Z. GRACE: a comprehensive web-based platform for integrative single-cell transcriptome analysis. *NAR Genomics and Bioinformatics*. 2023;5(2):lqad050. doi:10.1093/nargab/lqad050

7. Yu G, Wang LG, Han Y, He QY. clusterProfiler: an R Package for Comparing Biological Themes Among Gene Clusters. *OMICS: A Journal of Integrative Biology*. 2012;16(5):284-287. doi:10.1089/omi.2011.0118

8. Jin S, Guerrero-Juarez CF, Zhang L, et al. Inference and analysis of cell-cell communication using CellChat. *Nat Commun*. 2021;12(1):1088. doi:10.1038/s41467-021-21246-9

9. Kumar N, Mishra B, Athar M, Mukhtar S. Inference of Gene Regulatory Network from Single-Cell Transcriptomic Data Using pySCENIC. In: Mukhtar S, ed. *Modeling Transcriptional Regulation*. Vol 2328. Methods in Molecular Biology. Springer US; 2021:171-182. doi:10.1007/978-1-0716-1534-8_10

10. Patel AP, Tirosh I, Trombetta JJ, et al. Single-cell RNA-seq highlights intratumoral heterogeneity in primary glioblastoma. *Science*. 2014;344(6190):1396-1401. doi:10.1126/science.1254257

**
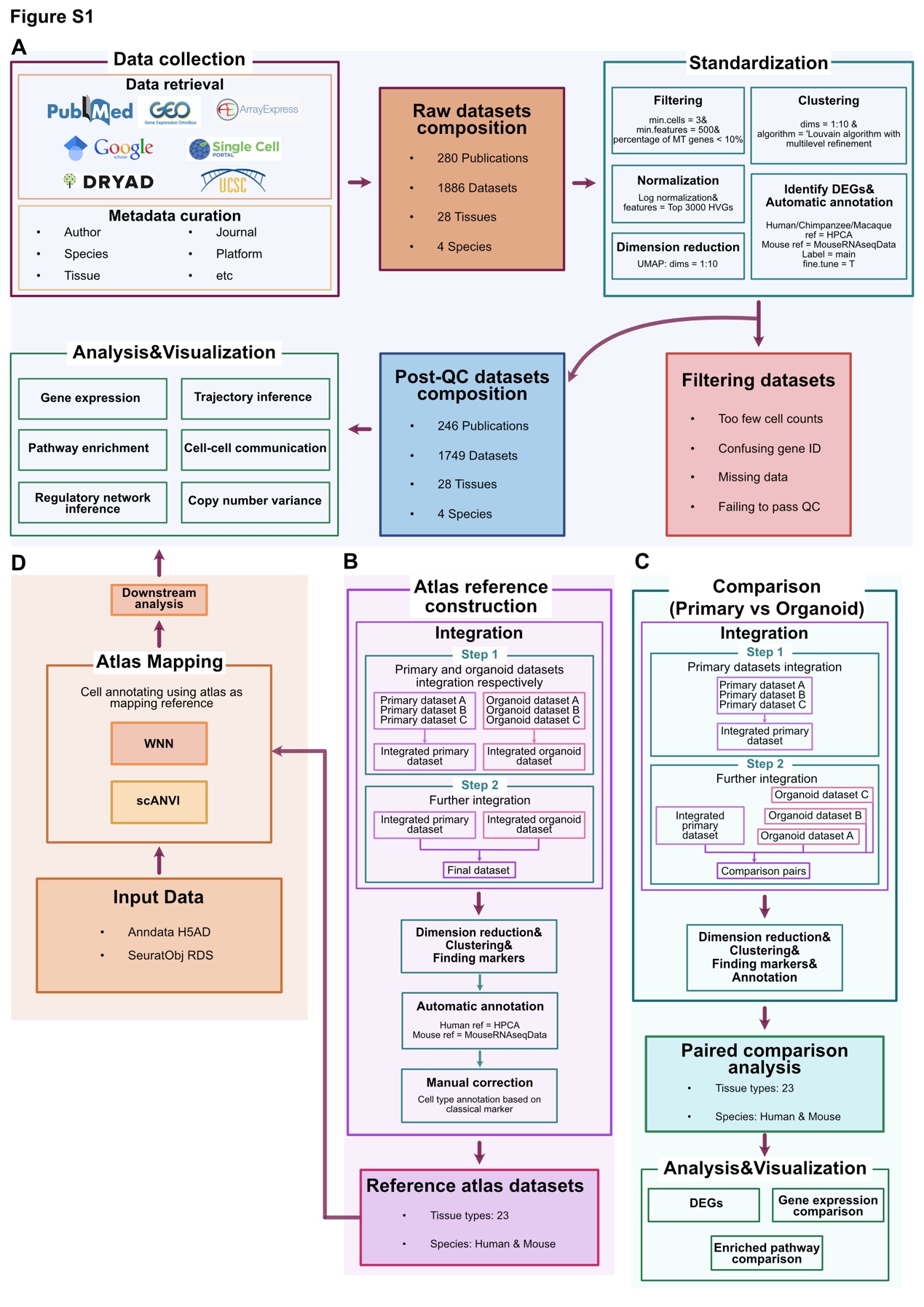
**

**Supplementary Figure 1.** (A) Diagram of data collection, pre-processing, analysis, and visualization. (B) Diagram of the construction of reference atlases for 23 tissues. (C) Diagram of the comparison between primary tissue and organoids. (D) The pipeline of one-stop data analysis module. Users’ data in the format of H5AD or RDS can be uploaded to OrganoidPortal for further analysis. High-quality reference atlases of 23 tissues are provided in the web server for cell clustering and annotation. The results of cell type classification and downstream analysis can be directly downloaded.


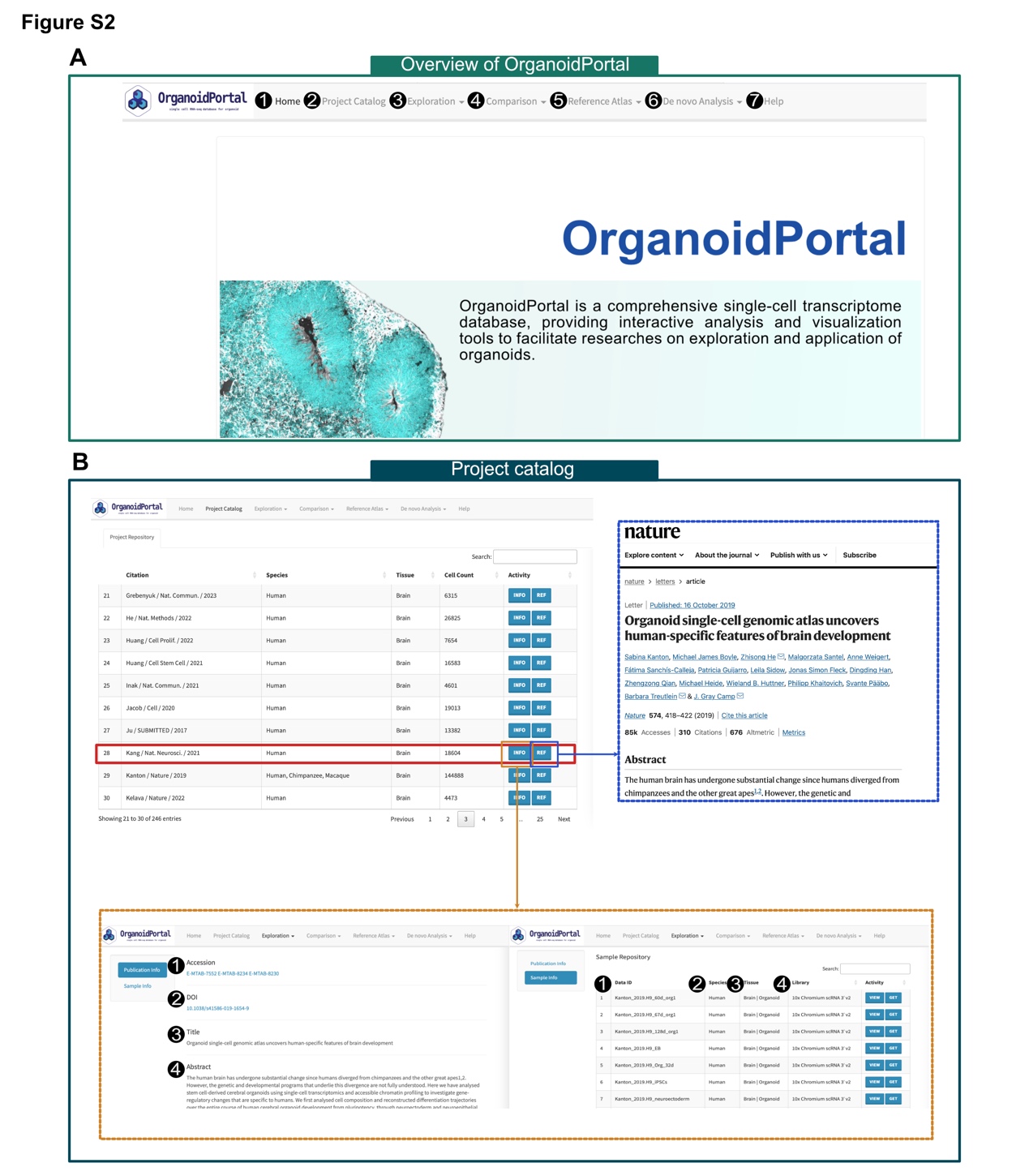


**Supplementary Figure 2.** (A) Snapshot of OrganoidPortal Homepage. (B) Construction and introduction of “Project catalog” module. “Project catalog” module provides information on data resources and queries, including article information and sample information.


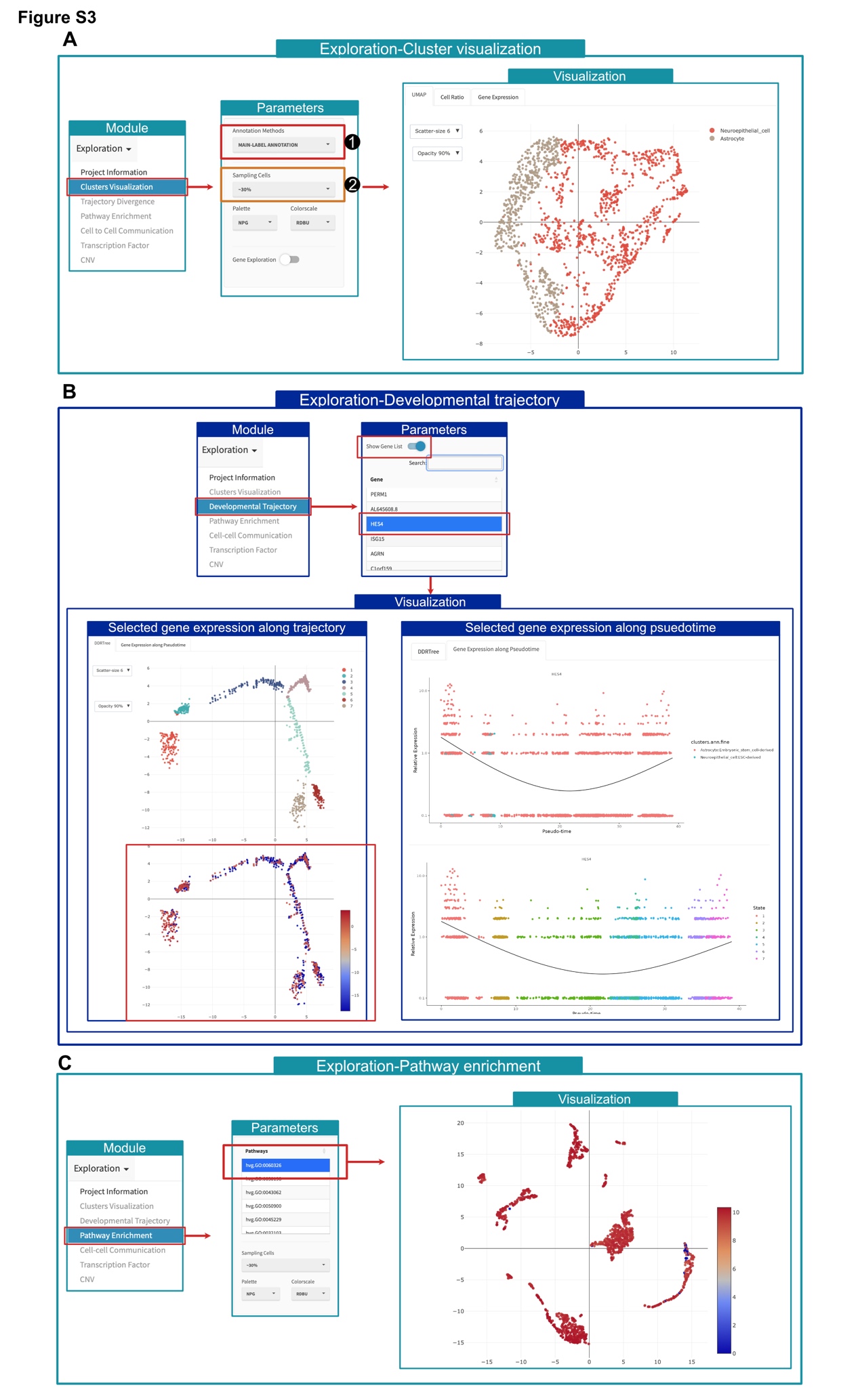


**Supplementary Figure 3.** In the module of “Exploration”, users can examine the visualization of (A) clustering, (B) developmental trajectory and (C) pathway enrichment.


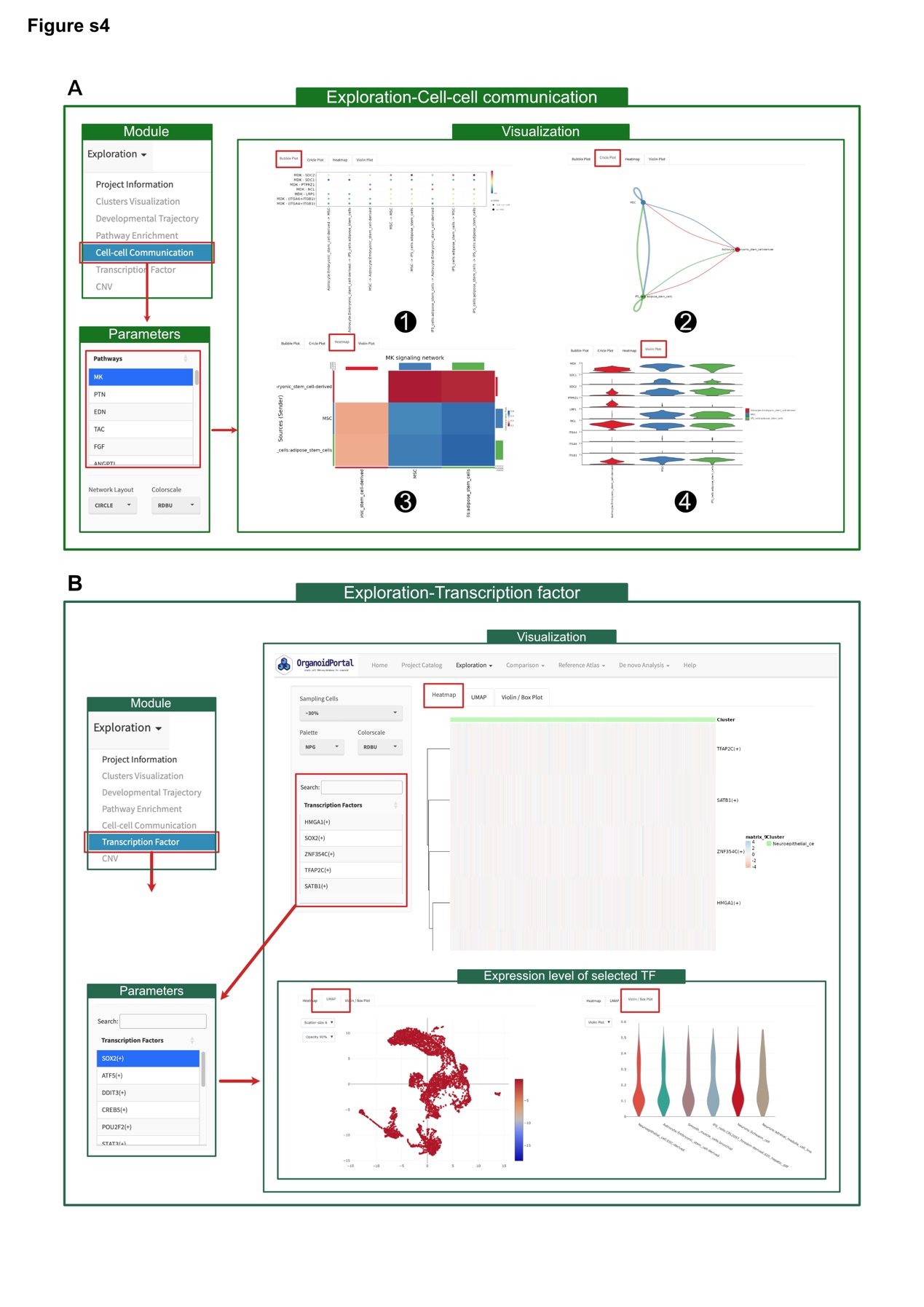


**Supplementary Figure 4.** In the module of “Exploration”, analyses of (A) Cell-cell communication and (B) Transcription factors are provided for data exploration.


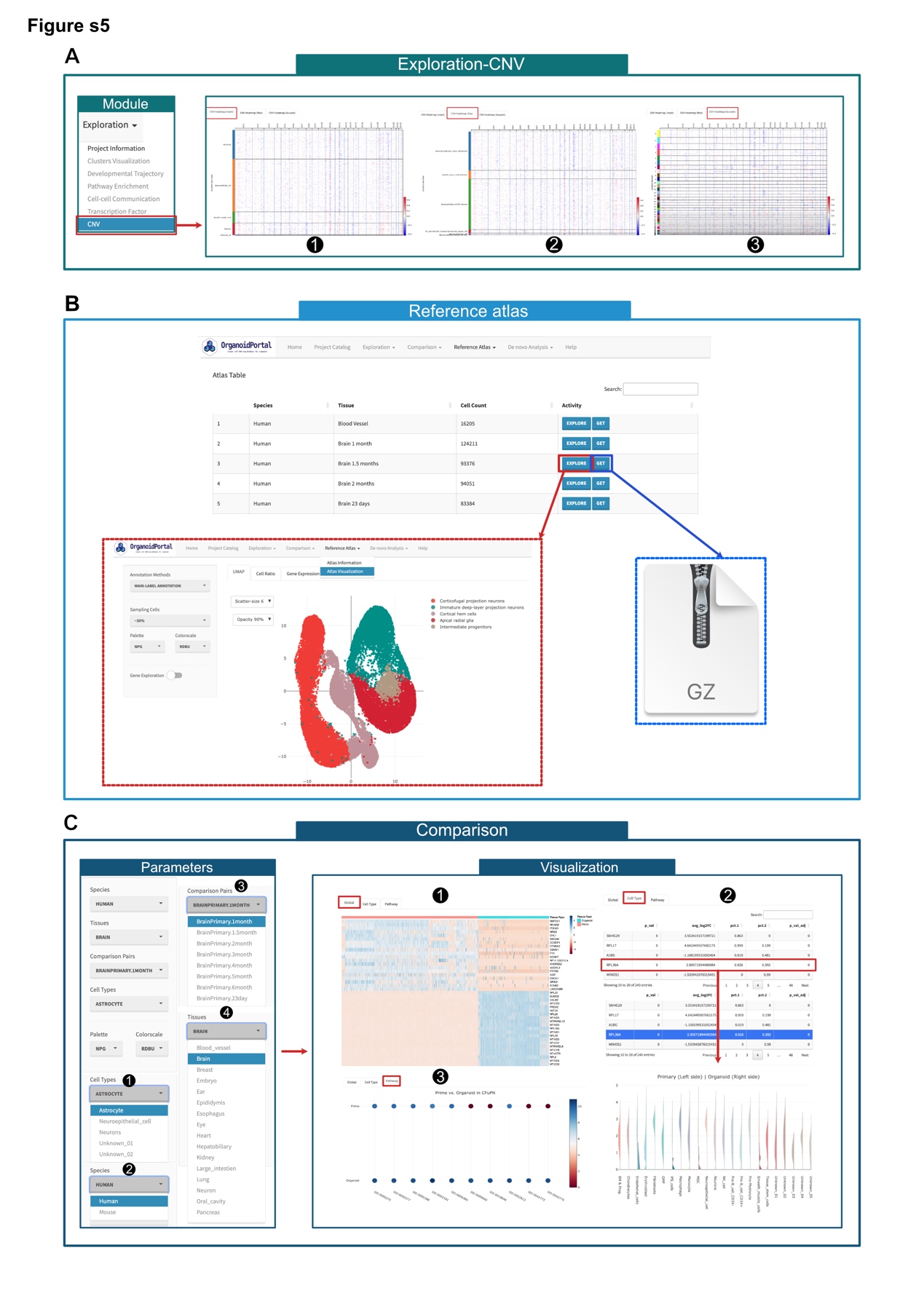


**Supplementary Figure 5.** (A) In the module of “Exploration”, heatmaps of copy number variation (CNV) analysis are visualized in terms of cell type annotation methods. (B) Information and functional analysis of reference atlas in the module of Reference Atlas. (C) The parameters and visualization in the module of Comparison between organoids and matched primary tissues.

**Supplementary Table 1.** Features of OrganoidPortal compared to other databases.

**Supplementary Table 2.** Information of technical and biological replicates obtained from original publication of reference atlas.
